# Warming-induced drought leads to tree growth decline in subtropics: Evidence from tree rings in central China

**DOI:** 10.3389/fpls.2022.964400

**Published:** 2022-09-23

**Authors:** Mengdan Jing, Liangjun Zhu, Shuguang Liu, Yang Cao, Yu Zhu, Wende Yan

**Affiliations:** ^1^National Engineering Laboratory for Applied Technology of Forestry and Ecology in South China, Central South University of Forestry and Technology, Changsha, China; ^2^College of Life Science and Technology, Central South University of Forestry and Technology, Changsha, China; ^3^Institute of Soil and Water Conservation, Northwest A&F University, Xianyang, Shaanxi, China

**Keywords:** tree rings, subtropical forests, rapid warming, tree growth decline, forest management

## Abstract

Subtropical forests provide diverse ecosystem services to human society. However, how subtropical tree species respond to climate change is still unclear. Using a dendrochronological method, we studied the radial growth patterns and species-specific responses of four main tree species in subtropical China to recent warming and drought. Results showed that the long-term drought caused by global warming and reduced precipitation since 1997 had resulted in the growth decline of *Pinus massoniana*, *Castanea henryi* and *Castanopsis eyrei* but not for *Liquidambar formosana*. Four species had similar sensitivities to the previous year and the current year, which is probably due to the carryover effect and temporal autocorrelation of climate data. Tree growth was positively correlated with growing season precipitation and relative humidity while negatively correlated with vapor pressure deficit. The negative relationship of tree radial growth with temperatures in the previous and current summer and the positive correlation with precipitation gradually strengthened after 1997. Therefore, we highlighted that drought-induced tree decline in subtropical forests is probably a common phenomenon, and it needed to verify by more tree-ring studies on a large scale. The species-specific responses of tree radial growth to climate change are not obvious, but they still should be considered in regional carbon balance and forest dynamics. Considering future climate change, species that are more drought tolerant should be considered as potential plantation species.

## Introduction

Forest ecosystems, an imperative part of the terrestrial ecosystem, account for 30% of the area of the global terrestrial ecosystems (FAO, 2015). Forests act as an important net carbon sink (2.4 ± 0.4 Pg C year^−1^) and therefore play a key role in the terrestrial carbon cycle (Pan et al., 2011; Gomes et al., 2021). Climate change directly affects forest ecosystems, particularly the capacity of forests to take up carbon (Allen et al., 2015; Zuidema et al., 2022). A variety of climatic and atmospheric changes, including rising temperature, warming-induced atmospheric drought, shifting rainfall patterns, and elevated atmospheric CO_2_, can have various impacts on tree growth (Panthi et al., 2020).

The impact of global warming on forest ecosystems is uncertain mainly because of the following two reasons. The first is the spatiotemporal heterogeneity of climate change (IPCC, 2021). For example, most studies on tree growth and climate relationships present positive responses to global warming in high latitudes/elevations and negative responses at lower ones (Piao et al., 2019; Zhou et al., 2021). Second, different species have different strategies to cope with climatic changes, and species specificity or forest type is a key factor that defines the growth-climate response patterns (Fan et al., 2009; Rahman et al., 2019). For example, coniferous and broad-leaved species often have different tree growth-climate relationships due to their distinct internal morphological traits, physiological mechanisms, and phonological processes (Dai et al., 2020). Therefore, more work and data on different species or climate conditions are needed to clarify the uncertainty in accessing the effects of climate warming on forest growth (Allen et al., 2015). At the same time, understanding the differences in the responses of different tree species to climate change is of great significance for future forest management and plantations.

Trees are widely distributed and climate sensitive, and their rings can be easily cross-dated (Fritts et al., 1969). As natural archives, tree rings are valuable sources for detecting tree growth-climate relationships, providing accurate proxies for paleoenvironmental studies from local to hemispheric scales (Rahman et al., 2019). Tree ring data have become increasingly valuable in disclosing the long-term climate dynamics and studying the relationship between global warming and forest ecosystems in different regions (Zhu et al., 2021). Hence, quantifying the tree growth-climate relationships using dendrochronological methods will help us understand how trees respond to climate change and hence improve predictions of forest dynamics under future climate conditions.

Subtropical forests are an important part of the world’s forests (Sharma et al., 2022). They are structurally complex, rich in species composition, and essential for the provision of diverse ecosystem services to human society (Isbell et al., 2015; Ferreira et al., 2018). The subtropical forest ecosystem is important for studying the global terrestrial ecosystem carbon cycle (Yu et al., 2014). The East Asian summer monsoon brings a high amount of precipitation to the subtropics of China, home of the world’s largest subtropical evergreen broad-leaved forest, which is known as the ‘oasis’ on the tropic of cancer (Cook et al., 2010). With rich species composition and diverse vegetation types, subtropical forests play a significant role in maintaining local and even global carbon balance and biodiversity conservation. Nevertheless, climate changes have severely impacted the composition and redistribution of species in subtropical forest ecosystems (Zhou et al., 2013). Specifically, substantial mortality of tree species and significant changes in community structure have been evident in subtropical forests in southern China over the past 60 years, which is highly correlated with warming and increased soil dryness (Zhou et al., 2013).

In the last decade, an increasing number of tree-ring studies have been carried out in subtropical regions (Liang et al., 2019; Su et al., 2021; Sharma et al., 2022; Yang et al., 2022a). Many studies conducted in subtropical regions ecosystems suggest that drought stress caused by regional warming has caused vegetation shifts (Allen et al., 2010), increased tree mortality (Allen et al., 2015), increased die-off of some species (Breshears et al., 2005), decreased tree radial growth (Sharma et al., 2022), and decreased carbon sequestration in vegetation (Brienen et al., 2012). These studies suggest that subtropical forests were threatened by their lack of resilience against long-term climate changes (Zhou et al., 2013). Although some dendrochronological studies have been carried out in the subtropical regions of China, dendroecology studies are still relatively incomplete in this area (Luo et al., 2017). Large-scale, multi-species assessments of subtropical China would allow us to better assess how environmental change may control forest growth and the functioning of this key ecosystem. Thus, new tree-ring chronologies of some key and widely distributed species that have not been studied previously from the subtropical zones of China are needed to identify and characterize the impact of climate change on tree growth, such as *Castanopsis eyrei*, *Liquidambar formosana*, and *Castanea henryi*.

Here, we carried out a dendrochronology study on four subtropical tree species (*Pinus massoniana*, *C. eyrei*, *L. formosana*, and *C. henryi*) growing in the northern Luoxiao Mountain in central China. We aim to (i) determine the growth trends of these tree species, (ii) reveal the differences in the growth-climate relationships of these tree species, and (iii) examine the temporal variation of climate-growth relationships of these tree species. We hypothesized that climate warming caused a decline in tree radial growth in subtropical regions. The growth-climate relationship has changed after climate warming, which differs among tree species.

## Materials and methods

### Study area and climate

**Figure 1:**
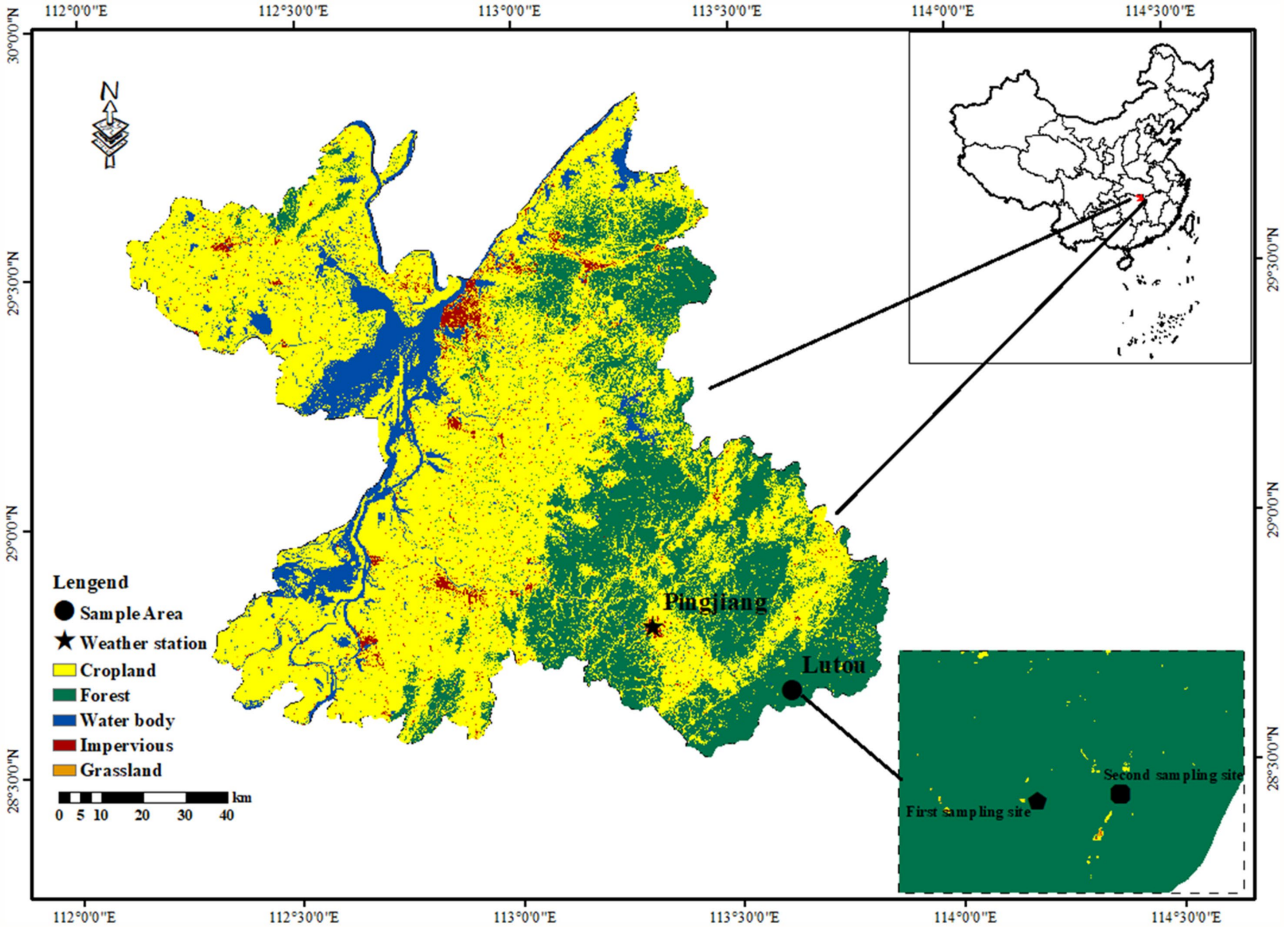
Location of the study area (Lutou) and weather station (Pingjiang) in the northern Luoxiao Mountain. Samples were collected at the first sampling site in September 2020 and at the second sampling site in July 2022.

**Figure 2:**
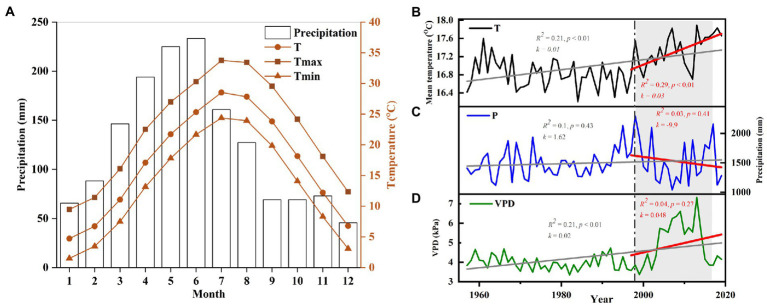
**(A)** Climograph of the study area showing monthly precipitation and temperatures from Pingjiang station during 1958–2019. Change trends of **(B)** mean temperature (T), **(C)** annual total precipitation (P), and **(D)** vapor pressure deficit (VPD). A linear regression equation fitted the interannual precipitation, temperature, and VPD trends from 1958 to 2019 (grey) and 1997 to 2019 (red). The *R*-squared, the slope (*k*), and the *value of p* of each model are listed in the figure. Severe drought periods are highlighted with a grey box.

The study was conducted at the Lutou National Station for Scientific Observation and Research of Forest Ecosystems (113°51′52″-113°58′24″E, 28°31′17″-28°38′00″N), Hunan Province, China (Figure 1). The elevation of the sampling sites ranges from 483 to 896 m. The forest communities in this area are mainly subtropical evergreen broad-leaved forests. The forest is mainly composed of *P. massoniana*, *C. eyrei*, *L. formosana*, *C. henryi*, *Rhododendron protist*, *Cyclocarya paliurus*, and *Cunninghamia lanceolata*. The study area is located in a subtropical humid continental monsoon climate zone with an apparent seasonal variability. Based on the instrument records of Pingjiang Weather Station (113°10′E, 28°43′N, 1097 m a.s.l.), the annual average temperature of the study area is 16.9°C, and the annual precipitation is 1,497 mm. January (1.53°C) and July (33.7°C) are the coldest and warmest months. Precipitation mainly occurs from March to July, accounting for 64% of the yearly rainfall. The frost-free period is about 260 days. The peaks of mean monthly temperature (July–August) and total monthly precipitation (May–June) are not synchronized (Figure 2). The Pettitt test shows that the climate in the region has changed significantly since 1997. The annual mean temperature (*k* = 0.01, *R*^2^ = 0.23, *p* < 0.001) and vapor pressure deficit (VPD; *k* = 1.62, *R*^2^ = 0.1, *p* < 0.001) in the study area showed significantly increasing trend. Especially after 1997, the annual mean temperature significantly increased at a rate of 0.03°C per year. The precipitation and VPD slightly decreased (*k* = −9.9, *R*^2^ = 0.03, *p* = 0.41) and increased (*k* = 0.048, *R*^2^ = 0.04, *p* = 0.27) after 1997. It is worth mentioning that the continuous high temperature and low precipitation from 1997 to 2019 led to a significant long-term drought in the study area (Figure 2).

### Sampling and data measurement

Based on reviewing relevant information and conducting a field survey of the study area, four common species (*C. eyrei*, *C. henryi*, *P. massoniana*, and *L. formosana*) were selected as target tree species. These species play an imperative role in the forest productivity and succession of forest communities in subtropical regions (Table 1). *P. massoniana* is a dominant local native tree species in south China. It is a fast-growing, evergreen, sun-loving pioneer conifer species, and a substantial increase in plantations of this species in south China has been witnessed during the past several decades. In contrast, *C. eyrei* and *C. henryi* are broad-leaved trees of intermediate shade-tolerance; *L. formosana*, a dominant deciduous tree species, has strong sprouting power and likes heat but is not drought tolerant. In October 2020 and July 2022, 10–50 mature and healthy individuals were selected as sampling trees for each species. One-to-two cores from each tree were collected at 1.3 m with a 5.15-mm diameter increment borer.

**Table 1:** Information of the sampling sites.

Species	Lat. (^o^N)	Long. (°E)	Ele. (m)	MRW (mm)	C/T	Time span	EPS	Rbar	MS	AC1
CE	28.56	113.92	750–889	3.24 ± 1.20	46/29	1890–2021	0.89	0.29	0.28	0.52
PM	28.56	113.92	483–889	1.49 ± 0.97	31/24	1882–2021	0.89	0.48	0.33	0.69
CH	28.56	113.92	757–889	2.57 ± 1.27	38/26	1899–2021	0.91	0.44	0.25	0.57
LF	28.54	113.93	868–896	3.19 ± 1.26	31/25	1941–2021	0.85	0.21	0.28	0.58

MRW, mean ring width (for whole series); EPS, expressed population signal; C/T, cores/trees; R_bar_, mean inter-series correlation; AC1, first-order autocorrelation; MS, mean sensitivity; CE, *Castanopsis eyrei*; CH, *Castanea henryi*; PM, *Pinus massoniana*; LF, *Liquidambar formosana*. The EPS, Rbar, MS, and AC1 were calculated from ring-width indices for the common period 1958–2019.

**Figure 3:**
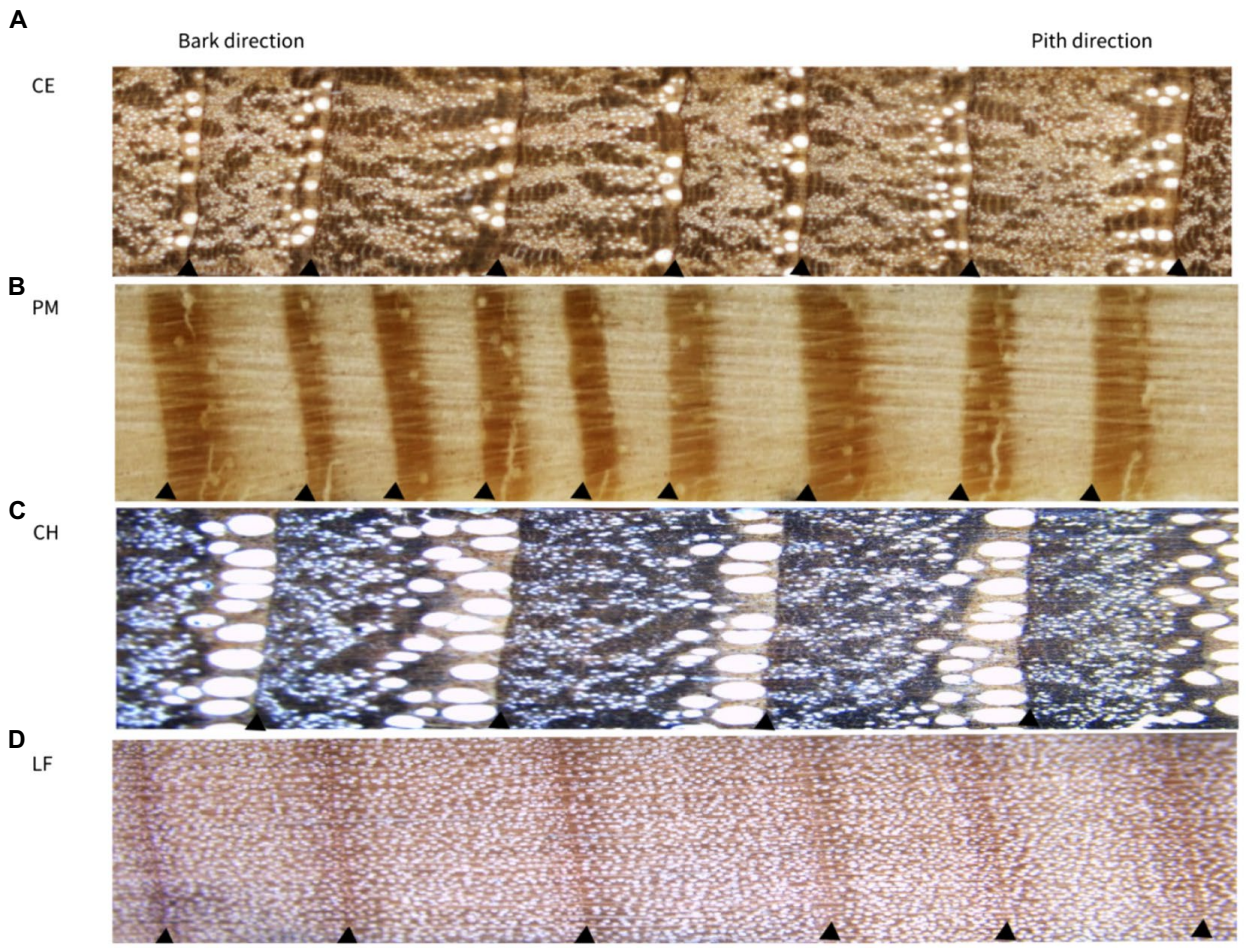
Photographs of the tree rings of the four tree species in the northern Luoxiao Mountain. **(A)** *C. eyrei*, **(B)** *P. massoniana*, **(C)** *C. henryi* and **(D)** *L. formosana*. The triangle indicates the ring boundary.

**Figure 4:**
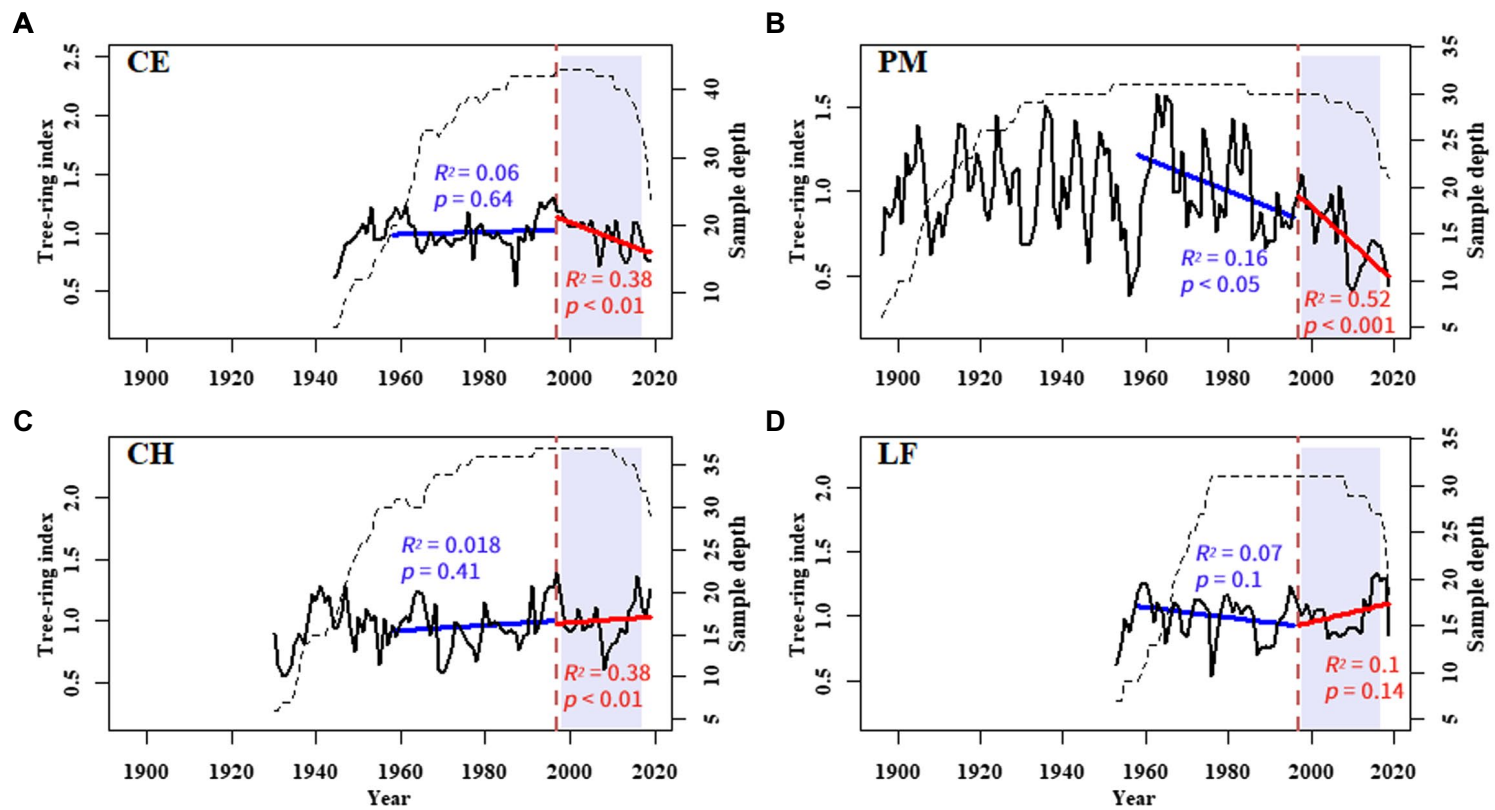
Tree-ring width standard chronologies of **(A)** *C. eyrei*, **(B)** *P. massoniana*, **(C)** *C. henryi*, and **(D)** *L. formosana* at the northern Luoxiao Mountain. The interannual growth trends from 1958–1996 (blue), and 1997–2019 (red) are fitted by a linear regression equation. The *R*-squared and *value of p* of each model are listed in the figure.

All tree-ring cores were fixed, dried, and polished in the laboratory (Figure 3). Then, they were visually cross-dated using the skeleton plot method under a binocular microscope (Stokes and Smiley, 1968). Tree ring width (TRW) was measured to an accuracy of 0.01 mm using a LINTAB™ 6 measuring system (RINNTECH, Germany). The quality of measurements and cross-dating were statistically tested using COFECHA (Holmes, 1983). The raw tree-ring series were detrended by fitting a negative exponential curve (ModNegExp) or a linear regression function with the “dplR” (Bunn, 2008) package in the R software (R Core Team, 2018). During this process, the conventional negative exponential curve or straight line of a negative slope or horizontal line was firstly applied to each tree-ring series. Then all the dimensionless indexes, computed by dividing the original measurement of each ring by the value of the fitted curve in the corresponding year, were combined into a single standard (STD) chronology (Figure 4) by computing a biweight robust mean (Cook, 1985).

To detect the growth trend of four species, we calculated the basal area increment (BAI) from tree ring width, assuming the increment of each ring is uniform in a circular cross-section of the tree (Peters et al., 2015). BAI is a more informative measure of tree growth trends and has been considered a stable form of growth (Phillips et al., 2009; Sharma et al., 2022). The negative BAI trend strongly indicates a decline in tree growth (LeBlanc, 1990). We sampled the trees to the pith; some cores were not taken perpendicular to the ring borders but with an arc on the cores if possible (Duncan, 1989; Rozas, 2003). In cores without a pith, the number of years missing to pith was estimated by a geometrical method. After dating the pith, we converted the measured tree-ring widths into BAI values using the BIAPlt software. TRW and BAI series were log-transformed and scaled to improve the normality of the data. We also developed regional curve standardization (RCS) chronologies by implementing pith offset. The division of measured values by the values of the age-related growth curve removes the age-related growth trend from measurements, removes the effects of overall differential tree growth rates, and results in a stationary series of tree indices which are in a suitable state to be averaged to form a chronology (Fritts et al., 1969).

### Climate data and statistical analysis

Instrumental climate data from the nearest Pingjiang weather station (~35 km) from 1957 to 2019 used in this study were downloaded from China Meteorological Data Service Center.^1^ Precipitation (P), relative humidity (RH), temperature (T), and extreme minimum (ETmin), extreme maximum (ETmax) temperatures at this station were extracted and used in this study. Atmospheric drought was quantified with the monthly VPD, calculated from the monthly mean temperature and relative humidity. Seasons were defined as follows: winter (December of the previous year to February of the current year), spring (March–May), summer (June–August), and autumn (September–November).

A linear regression model was used to fit the trends in tree growth or climate variations. Pearson correlation analysis was also carried out to determine the radial growth of the four tree species and the main monthly/seasonal climate factors. The abrupt point of climate change was tested using the Pettitt test in python software (Pettitt, 1979). Change-point, or step-trend, detection is an active area of research in statistics because change points may be evidence of natural or anthropogenic changes in climate (Ryberg et al., 2020). The Durbin-Watson test examined the first-order autocorrelation of climate data (Melo, 2014). Considering the lag effect of climate on tree growth, we used the climate variables from March of the previous year to November of the current year for monthly response analysis. Moving correlation analysis with a 25-year window was used to investigate the temporal stability of the relationships between radial growth and the main climate factors. Correlation analysis and moving correlation analysis were performed using the package “treeclim” (Zang and Biondi, 2015) in the R software.

## Results

### Growth characteristics of the four tree species

The correlation matrix between four species’ chronologies in the common period of 1958–2019 is shown in Table 2. The STD chronology of *L. formosana* was positively correlated with the STD chronology of *C. eyrei* (*R* = 0.297) and *C. henryi* (*R* = 0.354). And the STD chronologies of the other three tree species were also positively correlated with each other but not significant (Table 2).

**Table 2:** Correlation coefficients (*R*) between four standard tree-ring chronologies during their common period 1958–2019.

	CE	PM	CH	LF
CE	1			
PM	0.056	1		
CH	0.178	0.114	1	
LF	0.297^*^	0.047	0.354^**^	1

CE, *Castanopsis eyrei*, CH, *Castanea henryi*, PM, *Pinus massoniana*, LF, *Liquidambar formosana*.

* *p* < 0.05;

** *p* < 0.01.

**Figure 5:**
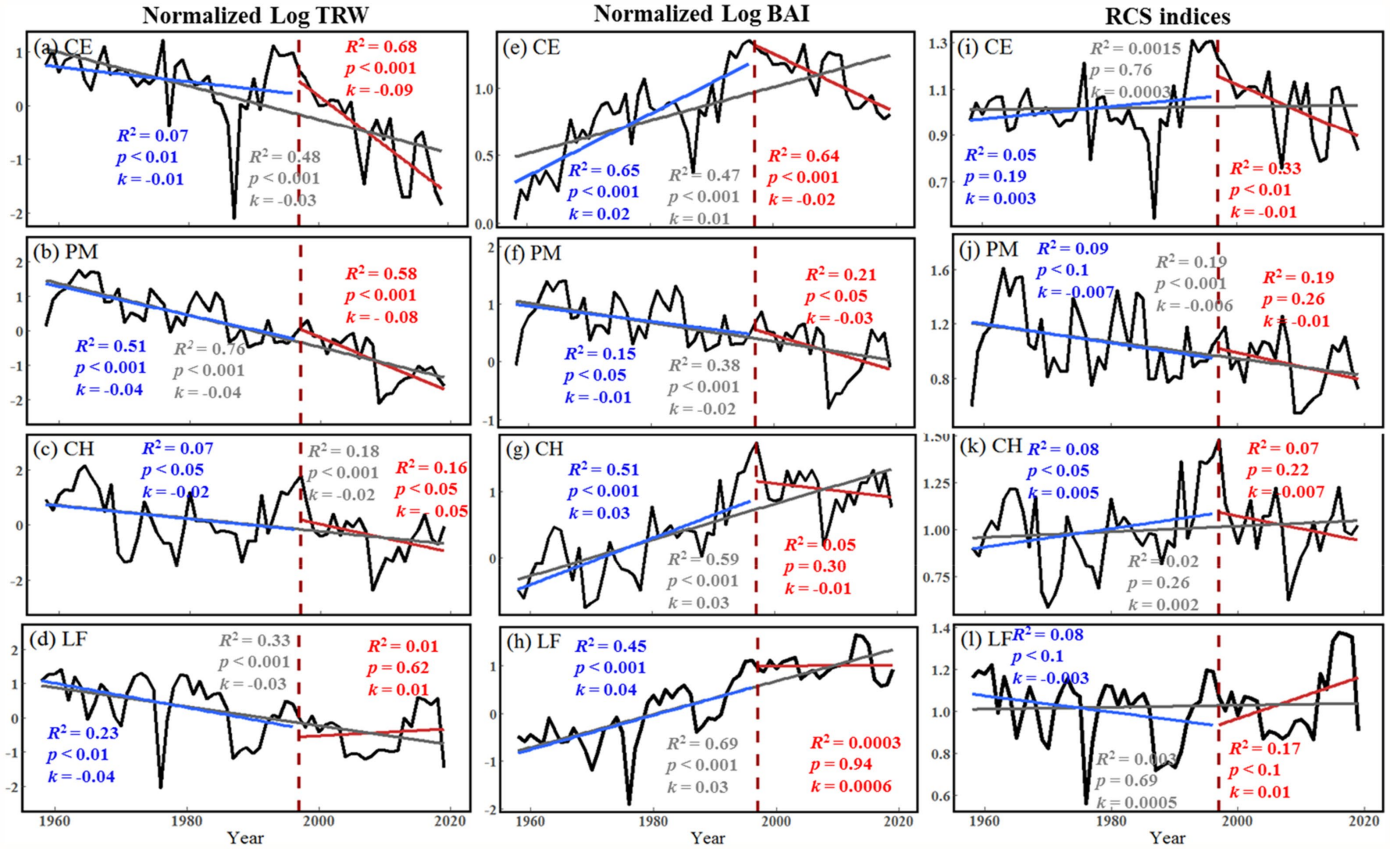
Long-term trends of tree growth (Normalized Log TRW, Normalized Log BAI, and RCS indices) during 1958–1996 (blue), 1997–2019 (red) and 1958–2019 (grey) at the northern Luoxiao Mountain. (a, e, i) CE = *C. eyrei*, (b, f, j) PM = *P. massoniana*, (c, g, k) CH = *C. henryi* and (d, h, l) LF = *L. formosana*. The *R*-squared, the slope (*k*) and *value of p* of each model are listed in the figure.

The normalized Log BAI and RCS chronologies showed similar trends (Figure 5). *C. eyrei* and *C. henryi* showed an increasing trend in 1958–2019 and 1958–1996. After 1997, the growth trend of *C. eyrei* and *C. henryi* changed, showing a downward trend. *P. massoniana* showed a significant downward trend in each growth period. The normalized Log TRW results showed that all tree species except *L. formosana* showed a decreasing trend in all growth periods. Normalized Log TRW results showed that *L. formosana* shows a weak growth trend (Figure 5).

### Relationships between radial growth and main climatic factors

**Figure 6:**
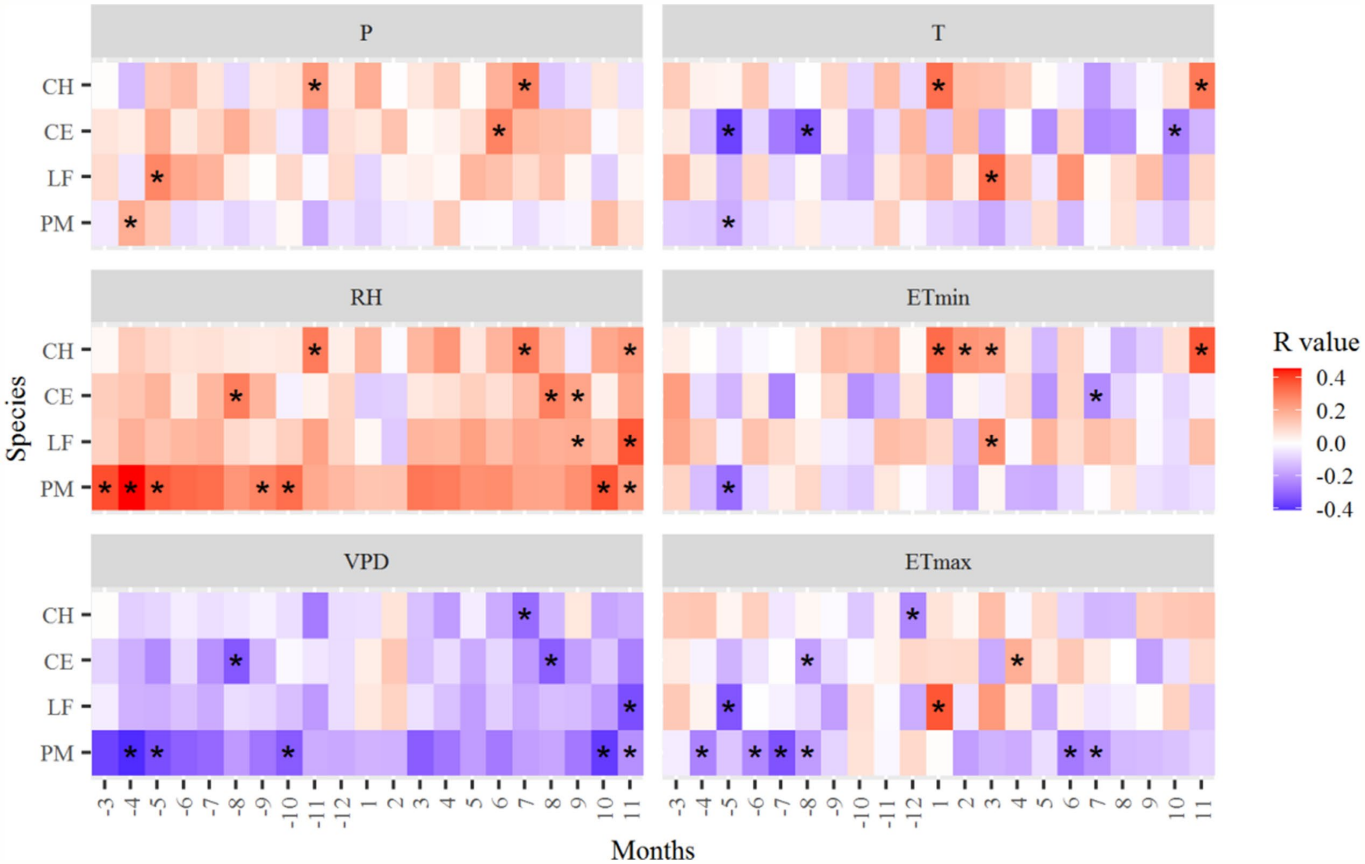
Correlation coefficients of the standard tree-ring chronologies in the northern Luoxiao Mountain with monthly main climate factors. P, precipitation, RH, relative humidity, VPD, vapor pressure deficit, T, mean temperature, ETmin, extreme minimum temperature, ETmax, extreme maximum temperature. Correlation analysis was computed for a 21 months window from previous year March to current year November. ^*^p < 0.05.

**Figure 7:**
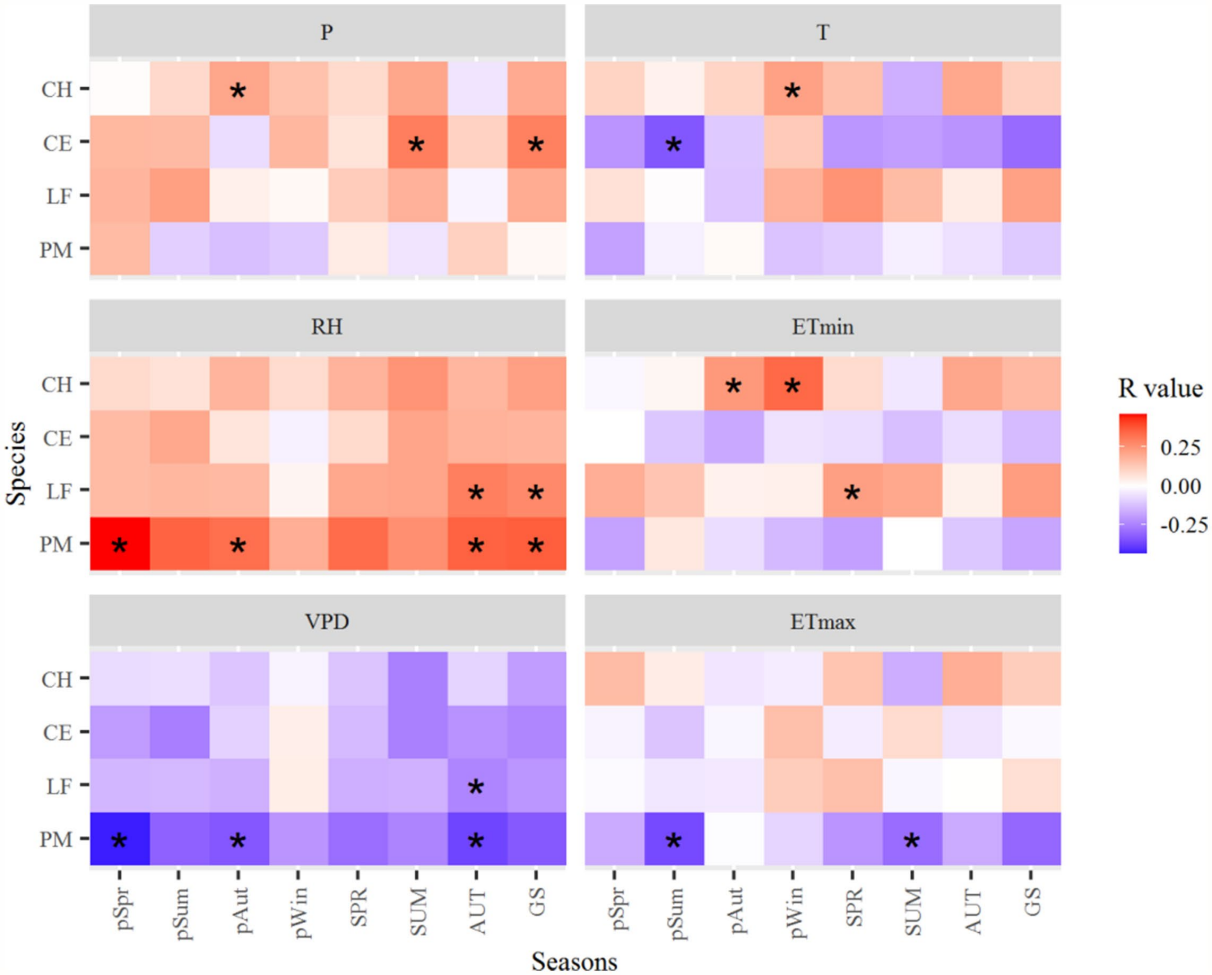
Correlation coefficients of the standard tree-ring chronologies in the northern Luoxiao Mountain with seasonal main climate factors during 1958–2019. P, precipitation, RH, relative humidity, VPD, vapor pressure deficit, T, mean temperature, ETmin, extreme minimum temperature, ETmax, extreme maximum temperature. pSpr, previous year March to May, pSum, previous year June to August, pAut, previous year September to November, pWin, previous year December to current year February, SPR, current year March to current May, SUM, current year June to August, AUT, current year September to November, GS, current year March to November. ^*^p < 0.05.

The relationships between radial growth and climatic variables differed among species (Figures 6, 7). The radial growth of all trees was mostly positively correlated with RH, and mostly negatively correlated with VPD. In the study area, moisture is the main climate factor limiting tree radial growth. ETmax was mostly negatively correlated with the growth of *P. massoniana*, especially in April–August of the previous year and June–July of the current year. However, ETmin and T in December of the previous year and November of the current year significantly positively affected the growth of *C. henryi*. P was most positively correlated with the ring-width chronologies of *C. eyrei*, *C. henryi*, and *L. formosana*, and negatively correlated with *P. massoniana* (Figure 6).

The growth of *C. henryi* significantly (*p* < 0.05) positively correlated with the P in the autumn of the previous year (Figure 7). The growth of *L. formosana* and *P. massoniana* significantly positively correlated with the RH in the autumn and growing season (March–November) of the current year. Radial growth of *P. massoniana* is negatively correlated with temperatures, especially the ETmax. The T in the summer of the previous year significantly negatively affected the growth of *C. eyrei*. However, the ETmin in the winter of the previous year and the autumn of the previous year positively affected the growth of *C. henryi* (Figure 7).

### Temporal stability in climate sensitivity of study species

**Figure 8:**
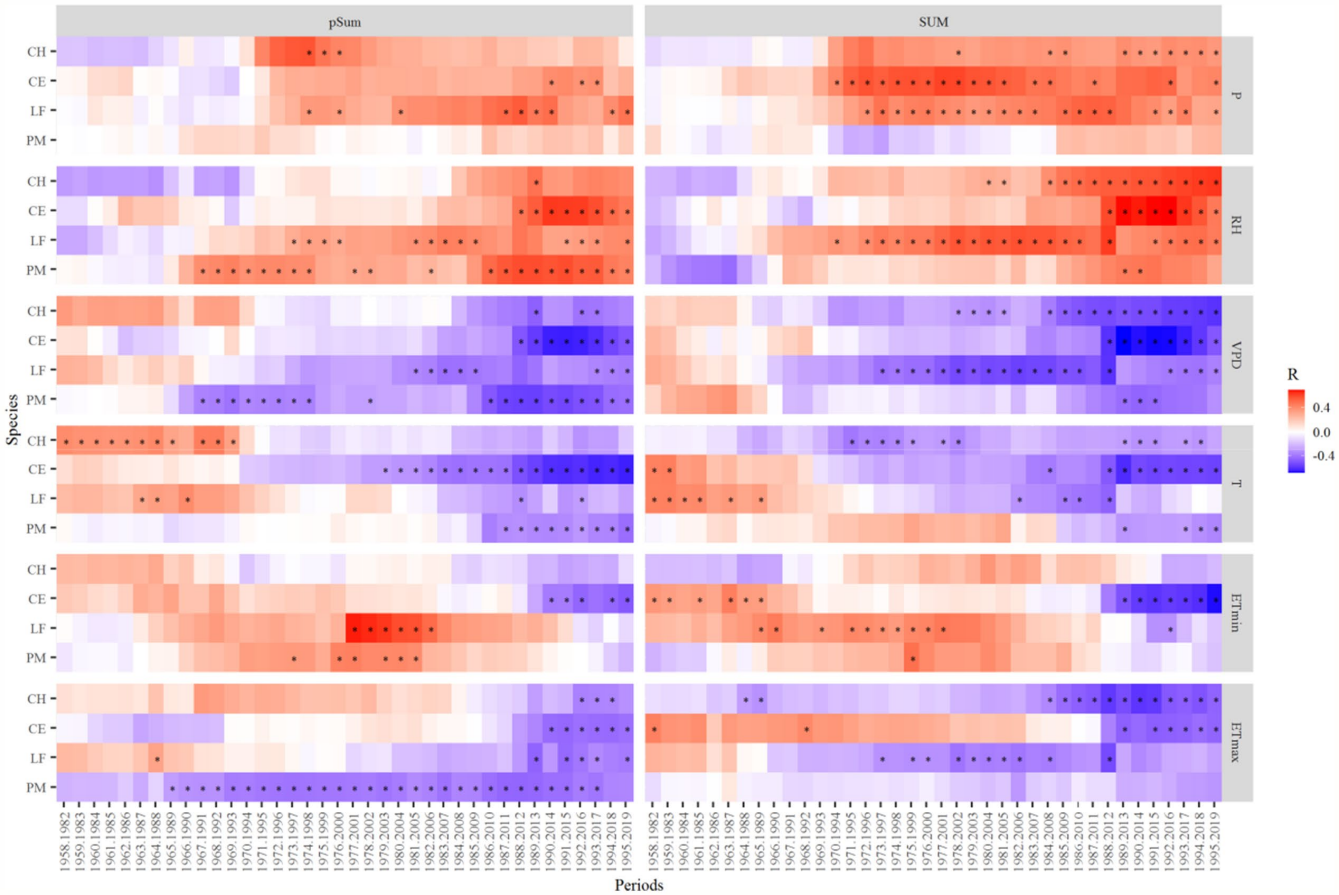
Moving correlation analysis of the standard tree-ring chronologies during 1958–2019. Correlation coefficients were computed for 25-year moving windows. The vertical bars on the right side of the graph represent the scale of correlation coefficients. pSum, previous year June to August, SUM, current year June to August. ^*^p < 0.05.

The growth responses of almost all tree species to the RH and P in the summer of the previous and current years changed from weak positive or negative correlation to strong positive correlation after 1997 (Figure 8). And the relationships between the radial growth of all species to T, ETmax, ETmin, and VPD in the summer of the previous and current years changed from a positive or weak negative correlation to a strong negative correlation (Figure 8).

## Discussion

### Tree growth decline in subtropical forest

All three analysis methods show the declining trend of radial growth of the three main tree species (Figure 5). Because of some potential bias, using growth rings to detect growth trends is not straightforward and should be considered to make accurate conclusions (Brienen et al., 2012). The “big tree sampling” bias is caused by sampling only the big trees, which usually results in positive growth trends over time. However, the “juvenile selection” effect often causes apparent negative growth trends (Brienen et al., 2012). Therefore, to detect and avoid the possible age/size-related spurious positive/negative trend, we used three different parameters: TRW, BAI, and RCS indices. The analysis results of RCS chronologies and normalized Log BAI showed that the “juvenile selection” did not play a very important role in the decline trend. The decline trend of tree radial growth after 1997 in this area is mainly caused by climate change.

That climate change could directly or indirectly initiate tree decline was first put forward by Hepting (1963). After that, the investigation of climatic influences on radial growth was widely undertaken to better understand the unexpected decline of trees, such as the *Pinus koraiensis* and *Picea jezoensis* in northeast Asia (Zhu et al., 2018; Wang et al., 2019), the *Nothofagus macrocarpa* and *Pseudotsuga menziesii* in North America (Klesse et al., 2020), and the *Abies alba* and *Fugus sylvatica* in Europe (Gazol et al., 2015; Bosela et al., 2018). Analysis of growth trends showed a declining trend in growth for most tree species after 1997 (Figure 5). It contradicts with our common belief that the subtropical forest ecosystems are expected to largely benefit or at least tolerate increasing temperatures (Boisvenue and Running, 2006; Allen et al., 2015). This general growth decline of subtropical trees in our study area is probably related to local climate change.

The radial growth of most subtropical trees was negatively correlated with VPD and positively correlated with relative humidity (Figures 6, 7). It suggested that water availability limits the radial growth of trees in subtropical regions. In the study area, there was a drying trend after 1997 due to significantly increased temperature and decreased precipitation (Figure 2). Hence, warming and increasing atmospheric drought at our study sites can lead to a decline in tree growth. Our results are consistent with studies by Su et al. (2021) and Huang et al. (2021), where drought contributed to the decline of forests in subtropical regions. Firstly, drought stress reduces leaf size, stem extension, and root proliferation, disturbs plant water relations, and reduces water-use efficiency (Farooq et al., 2009; Allen et al., 2015). Moreover, warming-induced high evapotranspiration reduces soil water content, vegetation photosynthesis, and carbohydrate reserves. At the same time, it will increase the atmospheric demand for water vapor, which deteriorates the water status in leaf and stem tissues (Wang et al., 2019).

In fact, the adverse effects of high temperatures and summer droughts on tree growth are widespread worldwide (Schwab et al., 2018; Harvey et al., 2020). Prolonged water deficits have caused increased tree mortality rates, severe forest decline, and vegetation shifts on dry sites and at the edge of species distribution ranges (Rigling et al., 2002; Galiano et al., 2010). For example, forest mortality has risen rapidly in temperate and boreal regions of southern Europe (Bosela et al., 2021; Martinez del Castillo et al., 2022) and western North America (Bigler et al., 2006; Bréda et al., 2006; Keen et al., 2022), and widespread death of many tree species in multiple forest types has affected well over 10 million ha since 1997 (Raffa et al., 2008). Our results indicate that further increases in forest decline and even tree mortality could be expected if climate warming continues, potentially driving the eventual regional loss of current subtropical trees or forests (Liu et al., 2020). A reduction in tree radial growth caused by warming-mediated drought stress has been found in many regions of China, such as tropical (Sharma et al., 2022), subtropical (Yang et al., 2022b), and Tibetan Plateau (Liang et al., 2016). The warming of the climate and the rise of atmospheric carbon dioxide increase the respiration of plants, leading to the reduction of carbon assimilation and inhibiting the radial growth of trees (Yang et al., 2022b). In addition, there is an urgent need for more studies at a regional scale to test the declining trends in subtropical forest growth and the mechanism behind it.

### The effect of climate change on the response stability of different tree species

Recent rapid warming in subtropical China has significantly affected tree radial growth. Moving correlation analysis showed that the negative correlation of tree growth with temperature and the positive correlation with precipitation gradually strengthened after 1997 (Figure 8). It is probably related to rapid warming in the study area. High summer temperatures can likely accelerate soil water evaporation and increase the VPD, thereby reducing soil moisture availability to trees (Keyimu et al., 2020). And higher temperatures exacerbate drought stress and increase the risk of drought-induced cavitation (Kannenberg et al., 2019). This suggests that the role of moisture availability in the growing season is becoming increasingly important for growth. Negative impacts due to increased temperature and moisture limitation will be more severe if climate warming intensifies. In the context of global warming, the effects of warm-induced drought stress on tree growth may be more severe, possibly leading to a large-scale tree decline in subtropical forests.

Climate change has been widely observed as a key factor that affects tree species’ sensitivity to climate (Kannenberg et al., 2019). Our results showed that tree growth-climate sensitivity is relatively consistent in this region, and tree species specificity is not obvious (Figure 8). This differs from other studies in subtropical regions. For example, Fan et al. (2009) pointed out that fir (*Abies georgei*) and spruce (*Picea brachytyla*) trees show a different climate-response behavior, both seasonally and in magnitude. The study of Yang et al. (2022b) in Southwest China showed that the two pine tree species (*Pinus kesiya* and *Pinus yunnanensis*) had different growth climate sensitivity. Growth-climate sensitivity of tree species varies with site-specific environmental conditions. The tree growth-climate sensitivity is not obvious among species in this study. It is probably because the precipitation in this area is relatively abundant compared with that in Southwest China. Therefore, it may be subject to less drought stress compared with other regions.

Our results show that all four species revealed almost similar climate sensitivity (although they differed in magnitude) for both previous year and current year climate (Figures 6–8). There are two possible reasons: The first one could be the carryover effect of reserve carbohydrates (in leaves) from previous years or the legacy effect of climate. The second one is the temporal autocorrelation in climate data and collinearity among climate variables. The main reason for the similar sensitivities in the previous year and the current year in T, P, ETmin, and ETmax is the legacy effects (Supplementary Figure S1; Table 3). Evidence for a lagged climate-growth response is commonly found in dendrochronological studies across the tropical and subtropical regions (Dünisch et al., 2003; Brienen and Zuidema, 2005). Water balance condition of the current year is critical to tree stem radial growth, and the pre-growing season droughts could profoundly affect later radial growth. Both the onset timing and severity of drought strengthen its “legacy effects,” which reduce trees’ resilience and make it harder for them to recover completely from drought (Gao et al., 2018). However, the DW test results showed that RH and VPD have significant first-order autocorrelation (Table 3). And the first-order difference data analysis results also proved this conclusion (Supplementary Figure S1). Therefore, except for the “legacy effects,” similar climate sensitivities in previous and current year RH and VPD may also be caused by the temporal autocorrelation of climate data (Supplementary Figure S1; Table 3). In tree rings, the physiology of trees serves as a basis for growth resources from previous years to be carried over a number of forthcoming years, resulting in tree-ring values that are depending on temporally adjacent values (Fritts et al., 1969). The main component of this autocorrelation comes from climate, which shows persistence through fluctuations and trends (Karl, 1988). Data autocorrelation may have an impact on long-term climate reconstruction, which should be considered in future research on paleoclimate reconstruction in subtropical regions.

**Table 3:** First-order autocorrelation coefficients for climate data.

	P	T	RH	VPD	ETmin	ETmax
January	1.95	1.93	1.27^*^	1.43^*^	1.97	1.87
February	1.86	2.25	1.79	1.84	2.23	1.75
March	1.91	1.66	0.85^*^	1.06^*^	1.70	1.67
April	2.09	1.95	1.09^*^	1.18^*^	2.52	2.25
May	2.09	2.06	1.06^*^	1.13^*^	1.87	2.06
June	2.63	1.87	0.92^*^	1.03^*^	2.03	2.05
July	1.98	1.85	0.99^*^	1.14^*^	1.98	1.90
August	2.03	2.11	1.41	1.63	2.26	2.54
September	2.09	1.64	1.75	1.74	1.74	1.39^*^
October	1.89	1.89	0.96^*^	1.00^*^	2.07	2.52
November	2.23	2.17	1.74	1.74	2.23	2.47
December	2.33	1.76	1.79	1.72	2.08	2.32

* means that the error has a first-order autocorrelation.

P, precipitation; RH, relative humidity; VPD, vapor pressure deficit; T, mean temperature; ETmin, extreme minimum temperature; ETmax, extreme maximum temperature.

In this study, we found that *L. formosana* still showed a higher growth trend after climate change (Figure 5). The species-specific tree growth patterns and tolerance to drought are closely related to these tree species’ physiological structures and traits because each wood type (e.g., ring-porous, diffuse-porous, and coniferous) differs in the structure, size, and spatial distribution of xylem conduits and the scaling of hydraulic properties with stem diameter (Meinzer et al., 2013). Previous studies have reported that the ability of trees to tolerate a dry environment is more important than the rapid transport of water (Lemoine et al., 2001). Our study of different tree species in subtropical forests also supports this view. Coniferous trees with a tight stomatal regulation such as pines (Klein et al., 2018) might display a lower resistance with increasing drought intensity. In contrast, vulnerability to drought-induced cavitation tends to be higher in ring-porous species (*C. henryi* and *C. eyrei*), because large-vessel cavitation yields a considerable loss in hydraulic conductivity (Hacke et al., 2006). These hydraulic properties depending on xylem anatomical features are linked to downstream stomatal regulation of transpiration (Lemoine et al., 2001; Bush et al., 2008). It is well established that some deciduous angiosperm species depend on sugar reserves to start secondary growth (Michelot et al., 2012). The later cambial activity of diffuse-porous (*L. formosana*) enables them to avoid using their stored carbohydrates for leaf flush. The stem improved the water balance of *L. formosana* (Köcher et al., 2013).

A significant reduction in tree growth during drought years, as found in this study and other studies across the subtropical region, may have important implications for the global carbon cycle. If climate warming intensifies, it could lead to a decline or death of drought-intolerant species. Reforestation, restoration, and a large-scale plantation establishment (afforestation) around the subtropics have been accepted as mitigation strategies of ongoing climate change impacts (Stocker et al., 2014; Rahman et al., 2019). Species selection is important for plantation design and depends on management objectives. Survival of different tree species will develop in a changing climate to compose an optimal species portfolio, leading to changes in forest composition, structure, and productivity. Considering future climate change, *L. formosana* should be selected as the main afforestation tree species. Additionally, the results highlight that it is important to protect relatively humid habitats, choose drought-tolerant tree species, implement water and soil protection measures during forest restoration, and take other management measures to maintain forest productivity in our study area.

## Conclusion

To examine the potential effects of climate change on forest growth of major tree species in subtropical forests, we investigated the response of radial growth of four major tree species in subtropical forests to climate change. Our results suggested that warming-related drought stress contributes to the decline of most tree species in subtropical forests. The radial growth of most subtropical trees was negatively correlated with vapor pressure deficit and positively correlated with relative humidity. The results of the moving correlation analysis showed that the negative correlation of tree growth with temperature and the positive correlation with precipitation and relative humidity gradually strengthened. Drought stress caused by rapid warming may be the main factor affecting the radial growth of trees in subtropical regions. And due to the influence of the legacy effect and the temporal autocorrelation in climate data, the four tree species in the study area have a similar growth-climate relationship. If climate warming continues, it will potentially drive the eventual regional loss of current subtropical trees or forests. Determining which tree species are more tolerant of climate change should be considered in future forest management.

## Data availability statement

All data used in the study can be found in the Supplementary material.

## Author contributions

All authors listed have made a substantial, direct, and intellectual contribution to the work and approved it for publication.

## Funding

The study was supported by the National Natural Science Foundation of China (42107476 and 31901241), the China Postdoctoral Science Foundation (2020M682600), the Research Foundation of the Bureau of Education in Hunan Province (20B627), and the Start-up Scientific Research Foundation for the Introduction of Talents in Central South University of Forestry and Technology (2020YJ012).

## Conflict of interest

The authors declare that the research was conducted in the absence of any commercial or financial relationships that could be construed as a potential conflict of interest.

## Publisher’s note

All claims expressed in this article are solely those of the authors and do not necessarily represent those of their affiliated organizations, or those of the publisher, the editors and the reviewers. Any product that may be evaluated in this article, or claim that may be made by its manufacturer, is not guaranteed or endorsed by the publisher.

## Supplementary material

The Supplementary material for this article can be found online at: https://www.frontiersin.org/articles/10.3389/fpls.2022.964400/full#supplementary-material

## Footnotes

^1^ http://data.cma.cn/
