# Supplementary material for: Warming-induced drought leads to tree growth decline in subtropics: Evidence from tree rings in central China
Source: Front Plant Sci. 2022 Sep 23;13:964400. doi: 10.3389/fpls.2022.964400 (PMC9539437; doi:10.3389/fpls.2022.964400)
Supplement: Supplementary file 1 [file Data_Sheet_1.zip › supplementary.docx]

**
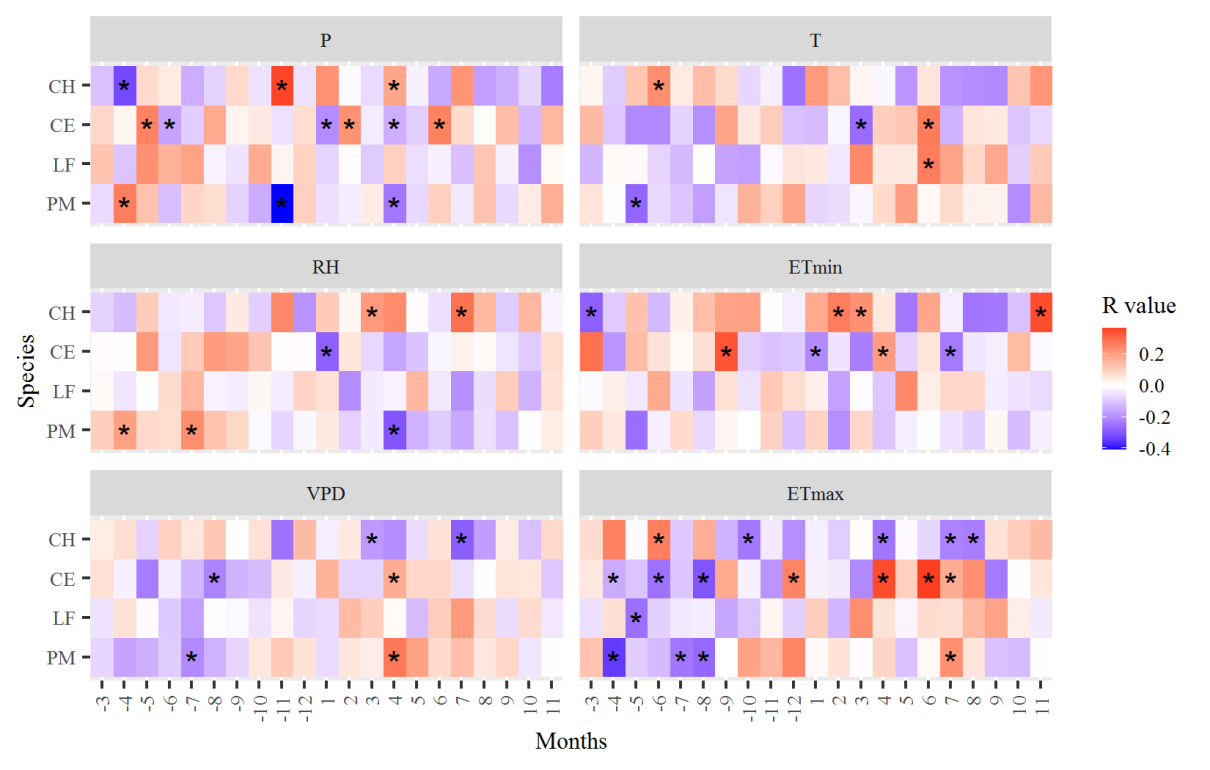
**

**Figure S1.** Correlation coefficients of the first-order differenced standard tree-ring chronologies in the northern Luoxiao Mountain with first-order differenced climate data. P = Precipitation, RH = relative humidity, VPD = vapor pressure deficit, T = mean temperature, ETmin = extreme minimum temperature, ETmax = extreme maximum temperature. Correlation analysis was computed for a 21 months window from previous year March to current year November. * = *p* < 0.05
